# Patient-derived organoids potentiate precision medicine in advanced clear cell renal cell carcinoma

**DOI:** 10.1093/pcmedi/pbac028

**Published:** 2022-12-06

**Authors:** Yizheng Xue, Bingran Wang, Yiying Tao, Jun Xia, Kedi Yuan, Junhua Zheng, Wei Zhai, Wei Xue

**Affiliations:** Department of Urology, Renji Hospital, Shanghai Jiao Tong University School of Medicine, Shanghai 200127, China; Ottawa-Shanghai Joint School of Medicine, Shanghai Jiao Tong University School of Medicine, Shanghai 200127, China; Ottawa-Shanghai Joint School of Medicine, Shanghai Jiao Tong University School of Medicine, Shanghai 200127, China; Department of Pathology, Renji Hospital, Shanghai Jiao Tong University School of Medicine, Shanghai 200127, China; Ottawa-Shanghai Joint School of Medicine, Shanghai Jiao Tong University School of Medicine, Shanghai 200127, China; Department of Urology, Renji Hospital, Shanghai Jiao Tong University School of Medicine, Shanghai 200127, China; Department of Urology, Renji Hospital, Shanghai Jiao Tong University School of Medicine, Shanghai 200127, China; Department of Urology, Renji Hospital, Shanghai Jiao Tong University School of Medicine, Shanghai 200127, China

**Keywords:** patient-derived organoid, air-liquid interface, tumor microenvironment, immunotherapy, renal cell carcinoma

## Abstract

To investigate the role of patient-derived organoid (PDO) model in the precision medicine of advanced clear cell renal cell carcinoma (ccRCC), we retrospectively analyzed the clinical data of seven cases of ccRCC diagnosed by operation and pathology in Renji Hospital from September 2021 to September 2022. The seven patients were diagnosed with advanced ccRCC with or without remote metastasis. Cytoreductive and radical nephrectomy was performed respectively. To predict the response to immunotherapy and provide personalized medicine recommendation, a PDO model based on air-liquid interface system was established from the surgical resected tumor and subsequent drug screening was performed. Hematoxylin and eosin (H&E) staining and immunohistochemistry revealed that the PDO recapitulated the histological feature of parent tumor. Immunofluorescence staining identified that CD3^+^ T cells, SMA^+^ cancer associated fibroblasts, and CD31^+^ endothelial cells were preserved in PDO models. Fluorescence activated cell sorter (FACS) revealed an evidently increased ratio of CD8^+^/CD4^+^ T cells and apoptotic tumor cells in PDO treated with toripalimab than those treated with IgG4. The results showed that toripalimab is able to rescue the excessive death of CD8^+^ T cells by critically reversing the immune exhaustion state of ccRCC in PDO model. This research validated that PDO is a promising and faithful preclinical model for prediction of immunotherapy response in patients with ccRCC.

## Introduction

Renal cell carcinoma (RCC) is the 6^th^ most common cancer in male, with poor prognosis due to its insensitivity to chemotherapy and radiotherapy.^1^ Clear cell renal cell carcinoma (ccRCC) is the most common pathological subtype of RCC, accounting for nearly 70% cases of RCC.^2^ Unfortunately, 25%-30% of RCC patients are in an advanced stage at primary diagnosis.^3^ For advanced RCC, the first-line treatment option recommended by the guideline is the multiple targets small molecule tyrosine kinase inhibitor (TKI) in combination with immune checkpoint blockade (ICB) or the combination of two ICBs.^4^ TKI inhibits cell growth and angiogenesis through blocking the signaling transduction downstream of vascular endothelial growth factor receptor (VEGFR), platelet-derived growth factor receptor (PDGFR) or blocking kinases related to cell proliferation and survival, such as c-Kit and Raf-1.^5–7^ ICB targets tumor infiltrated lymphocytes and reverses the immune exhaustion state,^8^ though with varied patient responses, due to tumor heterogeneity.^9^ Despite the discovery of some prognostic factors of ICB,^10–12^ the recent guidelines of RCC from EAU or NCCN failed to achieve precision medicine in this cancer.

Patient derived organoid (PDO) has emerged as a faithful personalized model for drug response prediction, which can be used in drug screening, clinical outcome prediction, and treatment strategy recommendation.^13–15^ Nevertheless, the conventional PDO model with only spheroid tumor epithelium fails to preserve tumor microenvironment (TME) and thus has limited application in RCC.^16^ Fortunately, a novel culture system of PDO based on air-liquid interface (ALI) has been reported to recapitulate both heterogeneity and architecture of TME,^17^ which provides new prospects for establishment of reliable preclinical model to predict responses to immunotherapy in patients with RCC.

Here, we report seven cases of advanced ccRCC with lung, liver, or bone metastasis. PDO models based on ALI system were established from the resected tissues in cytoreductive or curative surgery. The PDO model successfully mirrored the histological feature and immune cells infiltration of ccRCC. We further evaluated the treatment response to toripalimab, an immunotherapy agent.

## Methods & materials

### Ethical approval

All procedures performed involving human participants were approved by the Ethics Committee of Renji Hospital, Shanghai Jiao Tong University School of Medicine and were in accordance with the ethical standards of Helsinki Declaration (as revised in 2013). All the donors signed an informed consent form.

### Sample collection and processing

Freshly excised tumor samples from patients who received radical or partial nephrectomy in Renji Hospital (Table 1) were kept in MACS tissue storage solution (Miltenyi) at 4°C upon harvest. Tissue samples were cut into pieces for downstream processing and organoid establishment.

**Table 1.** Clinical information and predicted response to PD-1 blockade of PDOs cohort.

Sample	Sex	Age	Grade	PDO culture	T stage	N stage	M stage	Predicted response to PD-1
ccRCC-01	M	58	IV	Success	3a	0	1	Responsive
ccRCC-02	M	68	II	Success	3a	0	0	Resistant
ccRCC-03	M	49	II	Success	3a	0	0	Responsive
ccRCC-04	M	70	II	Success	3a	0	0	Resistant
ccRCC-06	M	69	III-IV	Success	1b	0	0	Responsive
ccRCC-07	M	72	II-III	Success	3a	0	1	Responsive
ccRCC-08	M	67	III	Success	4	0	1	Resistant

### PDO culture

Collagen gel matrices were prepared for transwell inserts by gently mixing collagen matrix (Cultrex Reduced Growth Factor Basement Membrane Extract, R & D Systems) and PDO culture medium (DMEM/F12 (lnvitrogen) containing 1X Normocin (Invivogen)), supplemented with N-Acetylcysteine (1mM Sigma), B-27 without vitamin A (1X Thermo), A83-01 (0.5µM, MCE), Y-27632 (10µM MCE), EGF (50ng/mL Peprotech), Nicotinamide (10mM, MCE), SB-202190 (10µM MCE), R-spondin-1, Noggin, Glutamax (1X, Gibco), Promocin (1X, Invivogen), IL-2 (600 IU/ml Novoprotein) on ice at a ratio of 1:100. Reconstituted collagen solution was added to the insert serving as a bottom layer gel. Then, the bottom layer was left to solidify at 37°C for 30 min, excess culture medium was aspirated.

After necrotic tissues and adipose tissues being removed, tumor tissues were minced into small pieces on ice, then washed twice using PDO culture medium followed by resuspended in 1mL of collagen matrix on ice. To form the double dish air-liquid culture system as described by Neal et al.,^17^ the collagen matrix with tumor tissues was added to the transwell insert, then placed into sufficient PDO culture medium within an outer cell culture dish. At last, the lid of the outer dish was replaced. Since one sample failed in PDO culture as the result of excessive necrosis with scarce viable cell left, 7 PDOs were established in the study.

### PDO drug test

Drug treatment started since the establishment of PDO culture system. Each insert was treated with toripalimab (10 µg/mL), or human isotype IgG4 (10 µg/mL) as negative control. Medium including tested drug was changed every 3 days. PDOs were grown for 1 week, then digested in collagenase D (1mg/ml in DMEM, Roche) for 30 mins to harvest single cell suspension for FACS analysis. The ratio of CD8^+^/CD4^+^ T lymphocytes was an indicator of salvage of exhausted immune microenvironment. Fixable viability stain 780 (BD Horizon^TM^) functioned as a marker of cell viability.

### H&E staining and IHC staining

Tissues and PDOs were processed into formalin-fixed, paraffin embedded (FFPE) blocks and sectioned (4–5 µm). Hematoxylin and eosin (H&E) E staining and immunohistochemistry (IHC) staining were performed following established staining protocols of routine laboratory.

### Immunofluorescence staining

Immunofluorescence stating was conducted on 4 µm sections of PDOs. After sections were dewaxed, rehydration and washed, they were incubated in boiled citrate buffer for 20 min for antigen retrieval. Slides were then blocked in 5% BAS in PBS and incubated with primary and secondary antibodies. Nuclei were stained with DAPI. Immunofluorescence images were acquired using a confocal microscope. Antibodies used were as follows: anti-CD3 (Abcam, 60181–1-Ig, 1:400); anti-SMA (Abcam, 67735–1-Ig, 1:400); Anti-CD31 (Abcam, 66065–2-Ig, 1:400).

### Multi-parameter flow cytometry and immunophenotyping

Fresh tumors and organoids were prepared into single cell suspension after digestion in collagenase D (1mg/ml in DMEM, Roche) at 37°C for 30 min. The samples were stained for 20 min at room temperature with a comprehensive antibody staining panel as follows: CD3 (BD Bioscience, 560 910), CD4 (BD Bioscience, 561 005), CD8 (BD Bioscience, 561 952), CD45 (BD Bioscience, 564 106), CD69 (BD Bioscience, 565 155), FVS780 (BD Bioscience, 565 388). The cells were washed with staining buffer before phenotyping. A total of 100 000 cells were collected using the Beckman Coulter CytoFLEX and analyzed with FlowJo (Version 10.8.1, Treestar).

## Results

### ccRCC PDOs preserve the histopathological feature of original tumors

**Figure 1.**
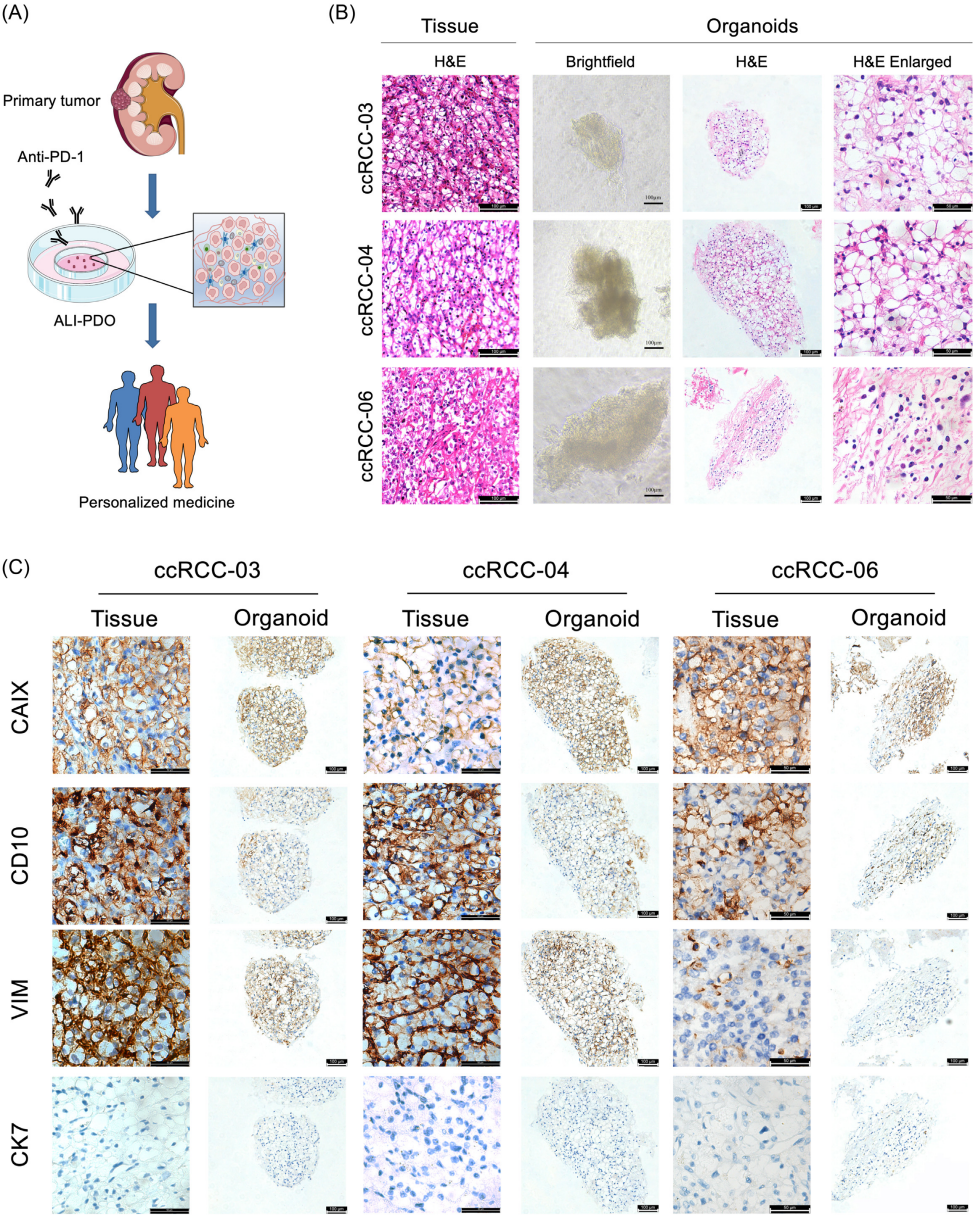
PDOs preserve histopathological features of original tissue. (A) Graphic abstract of experimental design; (B) Representative H&E staining images of ccRCC original tumors, together with light microscopy, H&E staining and enlarged images of corresponding PDOs; (C) Representative IHC staining images of matched original tumors and PDOs for CAIX, CD10, VIM, and CK7. PDOs, patient-derived organoids; H&E, hematoxylin and eosin; ccRCC, clear cell renal cell carcinoma; IHC, immunohistochemistry; CAIX, carbonic anhydrase IX; VIM, vimentin; CK7, cytokeratin.

To establish preclinical model for ccRCC, we successfully generated 7 PDOs from 8 surgical resected samples (87.5%). One sample failed as the result of excessive necrosis with scarce viable cell left. The tissue for cultivation was obtained from patients diagnosed with advanced ccRCC in Renji Hospital from September 2021 to September 2022 (Table 1). PDOs were established based on ALI system following the protocol established by Neal et al.^17^ In 7 of the PDOs, immunotherapy was provided to predict the response of patient to ICBs. Since our PDOs were established as a preclinical model to test the drug response under establishment for timely personalized medicine recommendation (Fig. 1A), we did not consider passaging or prolonged preservation.

We first examined whether the PDOs recapitulate the histopathological characteristics of parental tumor through H&E staining. Our results revealed that PDOs typically maintained the histopathological structures of parental tumor. For example, ccRCC-04_PDO showed classic clear cytoplasm resulting from the accumulation of glycogen and lipid (Fig. 1B). Then, IHC staining further confirmed the originality of PDOs. Carbonic anhydrase IX (CAIX), a specific marker of ccRCC was positively stained in all PDOs and matched tumor tissues. The expression levels of CD10 and Vimentin were preserved in PDOs and corresponding tumor tissues. All of the PDOs scarcely expressed cytokeratin 7 (CK7) (Fig. 1C). These results showed that each PDOs preserved the histopathological feature of original tumor.

### ccRCC PDOs maintain the composition and architecture of tumor microenvironment

**Figure 2.**
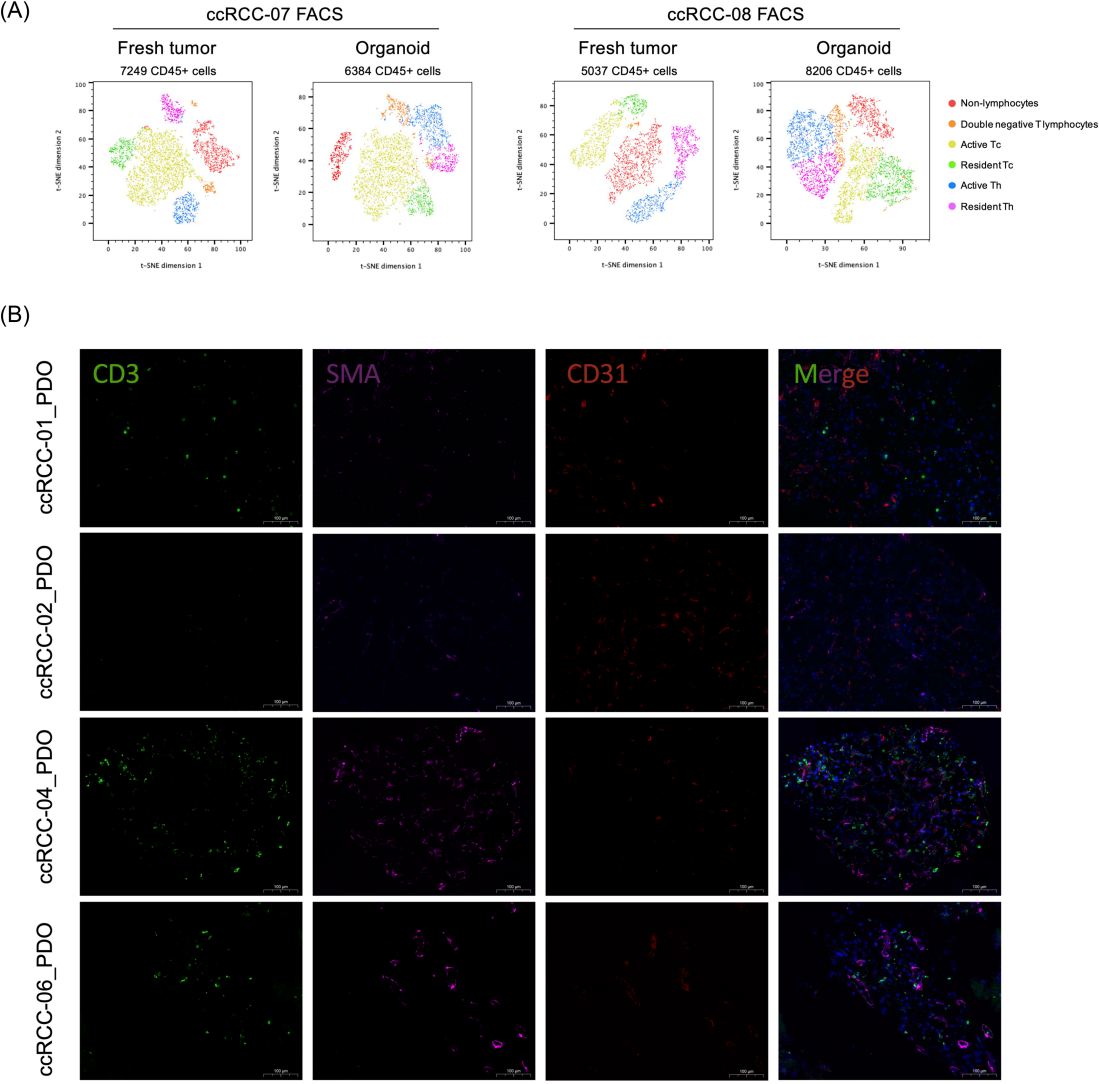
PDOs maintain the composition and architecture of tumor microenvironment. (A) t-SNE plots of multi-parameter flow cytometry and immunophenotyping of CD45^+^ immune cells from fresh tumor (left) and paired PDOs at day 7 (right); (B) Immunofluorescence staining images of ccRCC-01, 02, 04, and 06_PDO at day 7 for CD3 (green), CD31 (red), and SMA (magenta). Nuclei were stained with DAPI (blue).

We next compared the tumor infiltrated immune populations between parental tumors and corresponding organoids at day 7. FACS analysis revealed that PDOs recapitulated the diverse CD45^+^ immune cell subpopulations (Fig. 2A). In matched parental tumor and PDOs pairs (ccRCC-07 and ccRCC-08), the composition and subsets of CD8^+^ T cells, CD4^+^ T cells were mostly maintained.

**Figure 3.**
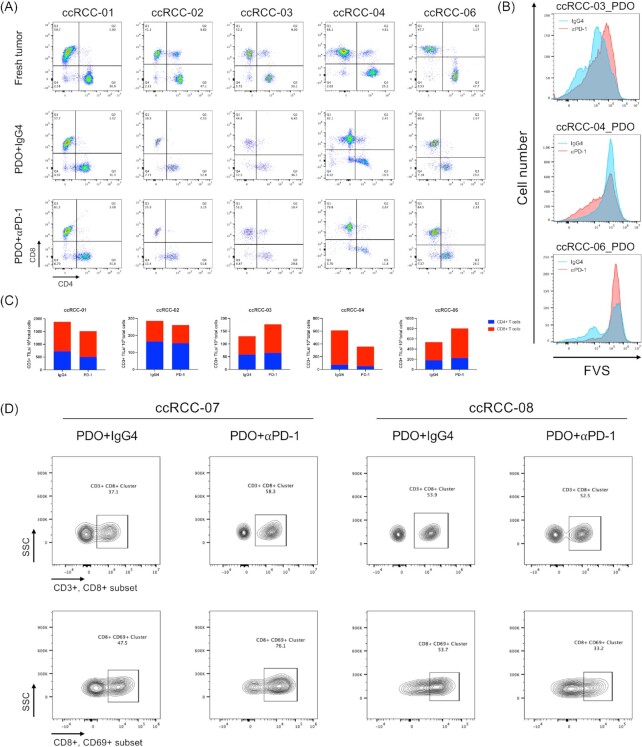
PDOs recapitulate the response to immune checkpoint blockades. (A) FACS analysis of tumor infiltrated CD8^+^ and CD4^+^ T lymphocytes subpopulations in original tumors (top row), and PDOs after 7 days treatment of toripalimab (middle row) versus isotype IgG4 (bottom row); (B) FACS histogram plot of FVS stained tumor cells in PDOs received toripalimab (red line) or IgG4 (blue line) for 7 days; (C) FACS quantification of CD3^+^ TILs, CD4^+^, and CD8^+^ T lymphocytes subpopulations from (A); (D) FACS analysis of tumor infiltrated CD3^+^CD8^+^ T lymphocytes and CD8^+^CD69^+^ T lymphocytes in PDOs after 7 days treatment of toripalimab versus isotype IgG4. PDOs, patient-derived organoids; FACS, florescence-activated cell sorting; CD, cluster of differentiation; IgG, immunoglobulin-G.

To further examine the architecture of TME in PDOs, immunofluorescence staining was performed. Our results confirmed that CD3^+^ T cells, SMA^+^ cancer-associated fibroblasts (CAFs) were preserved in PDOs (ccRCC-01 and ccRCC-02). CD31^+^ staining showed that endothelial cells formed neovasculature in PDOs, indicating that PDOs preserved the architecture of TME (Fig. 2B). Recently, Clark et al. defined 4 subtypes of ccRCC according to the TME components.^18^ Consequently, we interrogated whether intertumoral TME heterogeneity was presented in PDOs. ccRCC-01_PDO and ccRCC-06_PDO showed abundant T lymphocytes infiltration in TME, which could be classified as CD8^+^ inflamed subtype and predicted to be responsive to immunotherapy. Even ccRCC-04_PDO showed abundant infiltrated T lymphocytes, there also existed large amount of CAFs, which can be classified as CD8^−^ inflamed subtype and predicted to be resistant to immunotherapy as the result of excessive T cell exhaustion induced by suppressive immune cells, such as CAFs. Compared with ccRCC-01_PDO, ccRCC-02_PDO showed evident endothelial cell infiltration and neovasculature formation, while scarce CAFs and T lymphocytes infiltration, which could be classified as VEGF immune desert subtype and predicted to be resistant to immunotherapy. Further drug sensitivity tests also confirmed the results predicted by TME heterogeneity (Fig. 3A, 3C). To simply sum up, our PDOs successfully preserved the tumor infiltrated immune components, architecture, and heterogeneity of TME.

### ccRCC PDOs recapitulate the response to immune checkpoint blockades

To explore the application of PDOs as preclinical models to predict responses to immunotherapy, drug sensitivity tests on PDOs were performed. After 7-day treatment of toripalimab (PD-1 monoclonal antibody, 10 *μ*g/mL) or isotype IgG4 antibody as negative control, we investigated the ratio of CD8^+^/CD4^+^ T cells and the effect of tumor killing by fixable viability stain (FVS). In 3 of 5 PDOs (ccRCC-01, 03, 06), an increased ratio of CD8^+^/CD4^+^ T cells was observed after toripalimab treatment compared with IgG4 treatment, indicating that ICBs rescued the excessive death of CD8^+^ T cells which critically reversed the immune exhaustion state of TME. However, the changes of CD8^+^/CD4^+^ T cells ratio were not evident between toripalimab group and IgG4 group in ccRCC-02_PDO and ccRCC-04_PDO, suggesting failure of expanding CD8^+^ T cells after anti PD-1 treatment (Fig. 3A, 3C).

In parallel to the changed ratio of CD8^+^/CD4^+^ T cells, FVS also revealed an increased number of tumor death in ccRCC-03_PDO and ccRCC-06_PDO after toripalimab treatment, exhibiting anti PD-1 dependent tumor killing. In comparison, ccRCC-04_PDO had less viable tumor cells in IgG4 group than in toripalimab group, indicating resistance to ICBs (Fig. 3B).

To further investigate the response to PD-1 blockade, we detected the expression level of T cell activation associated marker: CD69.^19,20^ In ccRCC-07_PDO, the expression level of CD69 in CD8^+^ T cells was significantly increased under toripalimab treatment, which was consistent with the increased ratio of CD8^+^ T cells, indicating the rescue of T cell exhaustion state in the presence of PD-1 blockade. However, toripalimab showed inadequate ability of reversing T cell exhaustion in ccRCC-08_PDO compared with ccRCC-07_PDO (Fig. 3D). Thus our research found ccRCC-07 responsive, while ccRCC-08 irresponsive to PD-1 blockade.

In conclusion, our results showed the heterogeneity of responses to ICBs. Both changes in ratio of CD8^+^/CD4^+^ T cells and FVS indicated that ccRCC-01, 03, 06 were responsive, while ccRCC-02 and 04 were resistant to toripalimab (Fig. 3C). In addition, our findings of response heterogeneity were concordant with recent predictive models of immunotherapy. As illustrated above, ccRCC-01 was identified as ICB responsive CD8^+^ inflamed subtype while ccRCC-02 was identified as ICB resistant VEGF immune desert subtype.^18^

## Discussion

In this work, we reported the application of PDOs in predicting immunotherapy effect for patients with ccRCC. We successfully established PDOs based on ALI system and optimized the procedures for ccRCC, which not only recapitulated the histological feature of ccRCC parent tumor, but also preserved the tumor infiltrated lymphocytes. Consequently, compared with traditional PDO models, ALI-PDO is a promising preclinical model for predicting response to immunotherapy, which is recommended as the first-line treatment option for patients with advanced ccRCC. Recently, Esser et al. evaluated responses of PDO to immunotherapy through investigating necrosis area and area of viable cells in H&E stained slides.^21^ In patients with ccRCC, Krishna et al. also reported that elevated level of CD8^+^CD69^+^ T cells after PD-1 blockade can be observed in responsive patients.^11^ In this research, we applied the ratio of CD8^+^ T cell subset among total CD3^+^ T cells, CD69^+^ T cell subset among total CD8^+^ T cells, and cell viability staining to evaluate the response to immunotherapy.

In other cancer types, such as colorectal cancer, the precision medicine is under the guidance of next generation sequencing (NGS), since the correlations between genetics alteration and drug response has been extensively studied.^22^ However, biomarkers predicting drug response in patient with ccRCC remains unknown,^23,24^ resulting in limited benefits of NGS in the precision medicine of ccRCC. Consequently, the PDO models should be considered as a choice for achieving precision medicine of ccRCC. Li et al. successfully established RCC PDOs and revealed diverse responses of organoids to chemotherapy and targeted agents.^25^ Kazama et al. used PDOs to test the response to several TKIs.^26^ However, they both failed to test the response to immunotherapy, since the first generation PDOs failed to preserve immune cells infiltration. However, the significance of immunotherapy is stressed in guidelines and recommended as first-line treatment for patients with metastatic RCC.^4,7^ Consequently, PDO models with intact immune cell infiltration is in urgent need. Here we reported PDO models based on ALI system successfully preserving tumor infiltrating T lymphocytes and recapitulating the effect of immunotherapy. In the future, ALI-PDO is hopeful to become an *ex vivo* model in predicting response to first-line treatments recommended by guidelines, and thus instructing the medication choice of patient.

In this article, the response to immunotherapy was indirectly evaluated via the ratio of CD8^+^ T cells clusters and markers of cell viability. Consequently, a more accurate and sophisticated drug response evaluation system should be developed. In addition, as the patient cannot tolerate immunotherapy, we failed to test the consistency of PDOs prediction and clinical outcomes. In the future, based on the optimized protocol of PDOs culturing system, more patients with advanced RCC will benefit from PDOs, and thus a larger cohort of RCC with comprehensive long-term follow-up will be established for verifying the drug screening results. Moreover, PDOs established from core needle biopsy will be more helpful for neoadjuvant therapy or systemic therapy in metastatic renal cell carcinoma.
